# Evaluation of an application for the self-assessment of lifestyle behaviour in cardiac patients

**DOI:** 10.1007/s12471-023-01835-7

**Published:** 2023-12-07

**Authors:** Wilhelmina F. Goevaerts, Nicole C. C. W. Tenbült—van Limpt, Yuan Lu, Willem J. Kop, Hareld M. C. Kemps, Rutger W. M. Brouwers

**Affiliations:** 1 Department of Cardiology, Máxima Medical Centre, Eindhoven/Veldhoven, The Netherlands; 2 Industrial Design, Eindhoven University of Technology, Eindhoven, The Netherlands; 3 Center of Research on Psychological Disorders and Somatic Diseases (CoRPS), Department of Medical and Clinical Psychology, Tilburg University, Tilburg, The Netherlands

**Keywords:** Healthy lifestyle, Cardiovascular diseases, eHealth, Self-assessment, Patient activation, Transtheoretical model of behaviour change

## Abstract

**Background:**

Currently, no uniform, well-validated and comprehensive lifestyle behaviour self-assessment instrument exists for patients with cardiovascular disease.

**Purpose:**

To evaluate the usability of a novel mobile application (LifeStyleScore) based on validated instruments for the assessment of cardiovascular risk behaviours. Secondly, the application’s acceptance by healthcare professionals (HCPs) and its association with improved patient activation and lifestyle behaviour was evaluated.

**Methods:**

In this single-centre, non-randomised observational pilot study, patients with coronary artery disease or atrial fibrillation entering cardiac rehabilitation (CR) completed the LifeStyleScore application, the Patient Activation Measure (PAM-13®), and the System Usability Scale (SUS) during the CR intake and after CR completion. A focus group interview was performed with the HCPs involved.

**Results:**

We analysed 20 participants, 3 of whom were women, with a mean age of 61.9 ± 6.7 years. The LifeStyleScore application was rated with a SUS score above average (> 68) before (69.6 ± 13.4) and after CR (68.6 ± 15.1). All HCPs (*n* = 8) found the application usable. Patient activation did not increase significantly after CR compared with baseline (62.0 ± 8.6 versus 59.2 ± 9.5, respectively, *p* = 0.28) and only physical activity levels improved significantly (2.4 ± 0.7 (standardised score) at baseline, 2.8 ± 0.4 after CR, *p* = 0.04).

**Conclusion:**

The LifeStyleScore application was found to be usable for patients receiving CR. Its use did not result in increased patient activation, and of the lifestyle behaviours only physical activity levels improved. Further research is needed to evaluate how such applications can be optimally incorporated in CR programmes.

**Supplementary Information:**

The online version of this article (10.1007/s12471-023-01835-7) contains supplementary material, which is available to authorized users.

## What’s new?

- This study shows that the LifeStyleScore application, an application for comprehensive assessment of lifestyle behaviours in patients entering cardiac rehabilitation, scored satisfactory (above average) regarding usability.
- Healthcare professionals considered the application to be a helpful tool that increases patients’ lifestyle behaviour awareness and engagement.
- Although usability was satisfactory, patient activation and most lifestyle behaviours did not improve significantly after use of the application.
- For future studies, the application could be improved by increasing the amount of self-assessment moments and delivering more specific, patient-tailored advice for behaviour change based on the patient’s motivation and stage of change.

## Background

Current cardiac rehabilitation (CR) strategies aim to improve cardiovascular risk factors and lifestyle behaviours through educational sessions, personal or group-based counselling and behaviour change strategies such as motivational interviewing [1]. However, despite participation in CR, significant numbers of patients remain overweight, are physically inactive and keep smoking [2]. These suboptimal long-term results of current CR programmes can be explained by several factors, including insufficient long-term guidance, low participation and high dropout rates and, more importantly, insufficient focus on sustainable behaviour change and self-management [3].

Low participation and high dropout rates in CR might at least partly be a result of low motivation and low patient activation in a significant number of patients. Patient activation is defined as patients’ knowledge, skills and confidence to manage their health [4]. Studies have shown that patients with low motivational factors, such as low intention and low maintenance self-efficacy, tend to drop out earlier from CR programmes [5–8]. Vice versa, higher levels of autonomy and self-determination have been demonstrated to promote participation and motivation [9]. To achieve higher levels of activation, better participation and lower dropout rates, the components of CR programmes could be tailored more to patients’ individual needs and preferences. As a result, patients’ motivation to change their lifestyle behaviour may increase by applying behaviour change theories that better suit each individual patient.

According to lifestyle behaviour theories, self-regulation is a central mechanism of action in lifestyle behaviour change [10]. Self-monitoring, goal setting and problem solving are behaviour change techniques to support self-regulation in patients. To effectuate these techniques, it is essential to have a detailed insight into a patient’s lifestyle behaviour. Such insights can be attained by accurately and reliably assessing lifestyle behaviour data, which should be accessible for both the patient and the healthcare professional (HCP). Moreover, these insights might provoke a discussion between the HCP and patient about motivational issues and the barriers that prevent patients from setting goals towards improving lifestyle behaviour. Additionally, this discussion can lead to improved disease understanding and health literacy, which facilitates behavioural change. Studies show that improving the level of health literacy can lead to a change in lifestyle behaviour, such as smoking cessation [11] and increased physical activity [12].

Taken together, tools that assess lifestyle behavioural data and effectuate behaviour change techniques in clinical practice could therefore assist both patients and HCPs in making and monitoring healthy lifestyle choices, promote health behaviour change and support maintenance of these healthy lifestyle behaviours [1]. Currently, however, no uniform and validated instrument is available for the comprehensive assessment of lifestyle behaviours in patients with cardiovascular disease. The main purpose of the present study was to evaluate the usability of a newly developed mobile application based on validated instruments (the LifeStyleScore application) for the assessment of risk behaviours relevant for cardiovascular disease. As a secondary purpose, an exploratory analysis was performed to evaluate whether use of the application as an integral part of the CR care pathway was associated with an increase in patient activation and improvement in lifestyle behaviour change.

## Methods

### Study design

A single-centre, non-randomised observational pilot study was conducted in Máxima Medical Center in Veldhoven and Eindhoven, the Netherlands. Patients referred for CR due to coronary artery disease (stable angina pectoris, acute coronary syndrome and/or after coronary revascularisation) or atrial fibrillation who met the inclusion criteria as described in Tab. 1 were asked to participate in the study.

**Table 1:** Inclusion criteria

Inclusion criteria	
*i*	Patients referred for cardiac rehabilitation following treatment for coronary artery disease (i.e., stable angina pectoris, acute coronary syndrome and/or after coronary vascularisation) or atrial fibrillation
*ii*	Age ≥ 18 years
*iii*	Able to speak and read the Dutch language
*iv*	Access to internet and a computer, tablet or smartphone
*v*	Willing and able to provide informed consent

**Fig. 1:**
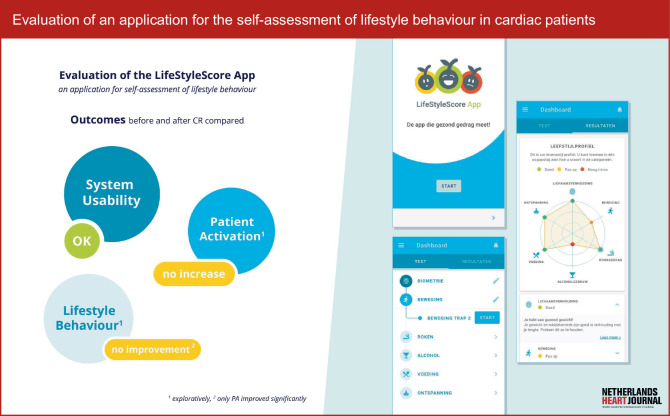
Infographic

**Fig. 2:**
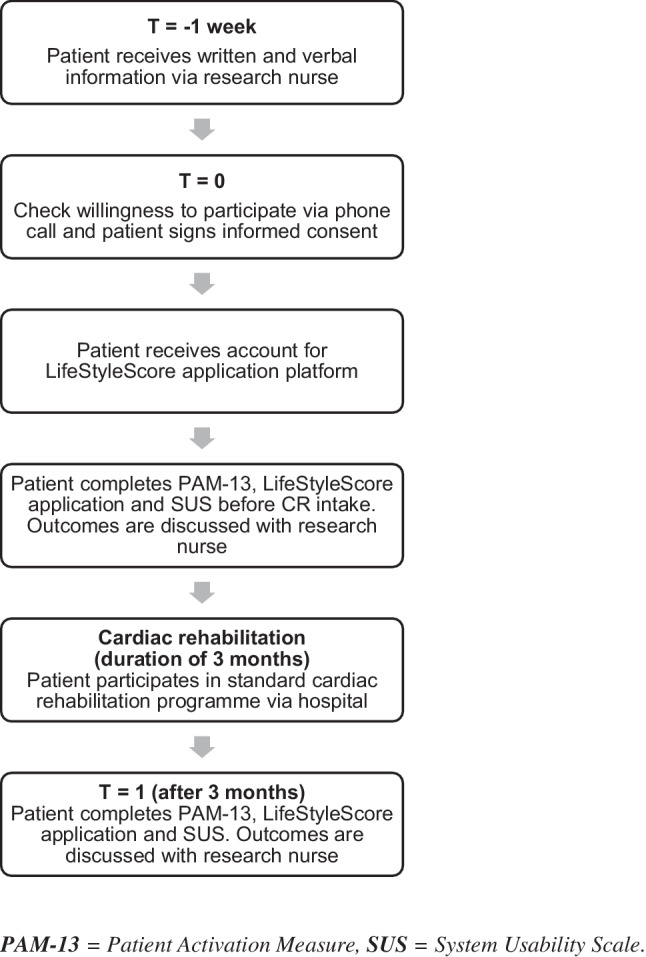
Study design

Recruitment was performed by a research nurse. After patients provided written and verbal informed consent an account for the LifeStyleScore research platform was created. The patient received a manual on how to access and complete the questionnaires in this study. Participants were instructed to complete the questions provided via the LifeStyleScore application as well as the Patient Activation Measure® (PAM®-13) [4] and the System Usability Scale (SUS) [13] at the start of their CR programme, i.e. before their first CR intake consultation with the case manager (t = 0), and after 3 months (t = 1), i.e. at the end of their CR programme. An impression of the LifeStyleScore application is displayed in Fig. 1. In order to assess patient activation tendencies prior to reporting actual lifestyle behaviours, the PAM-13 was administered first. Results of the LifeStyleScore application were used to set and evaluate lifestyle goals during the CR programme. An overview of the study design is displayed in Fig. 2.

### LifeStyleScore application

The LifeStyleScore application is based on validated assessment tools and provides insight into cardiovascular risk behaviour by assessing, scoring and giving advice in six separate lifestyle behaviour domains based on the Dutch vitality guidelines (BRAVO-kompas) [14] and a systematic review of the literature [15]. This entails that the application assesses physical activity, sedentary behaviour, smoking behaviour, alcohol consumption, nutritional intake and perceived stress, supplemented with biometric characteristics (body composition) of the patient. The first version of the application examined in this project is available in Dutch.

The assessment of lifestyle behaviour domains (except for body composition) encompasses two stages:

- *Stage 1*: a screening questionnaire used to detect cardiovascular risk behaviours;
- *Stage 2*: in case of possible cardiovascular risk behaviour, an in-depth questionnaire is used to more thoroughly evaluate the risk behaviour.

The patients received written feedback via the application based on their score. Cardiovascular risk behaviour scores are given as a traffic light score with the following categories: ‘Low risk’ (3—green), to ‘Medium risk’ (2—yellow) and ‘High risk’ (1—red).

For this study, the LifeStyleScore application was integrated into the CR programme. The patients filled out the application before their intake with their CR case manager. During the patients CR intake consultation, the results were discussed (motivational interviewing was used to motivate the patients if necessary) and used to formulate goals according to the SMART principle (Specific, Measurable, Achievable, Relevant and Time-Bound). The set goals were registered in the electronic patient record for follow-up. The cases were shared with other HCPs in the CR team. The questionnaires implemented in the LifeStyleScore application are based on validated questionnaires and guidelines as described in Tab. 2.

**Table 2:** Questionnaires used in the LifeStyleScore application

Lifestyle domain	Stage 1	Max. number of questionnaire items	Stage 2	Max. number of questionnaire items
Body composition	Gender, age, height, weight, body mass index (BMI) and waist circumference	5	N/A	
Physical activity	Habitual physical activity and sedentary behaviour (based on Dutch Physical Activity Guidelines [16])	2	International Physical Activity Questionnaire (IPAQ long form) [17]	25
Sedentary behaviour	Habitual sedentary behaviour (based on Dutch Physical activity Guidelines [16])	2	‘Wat beweegt jou’-questionnaire [18] (Only the ‘Hoeveel zit jij’-section)	8
Smoking cessation	Smoking status (average number of cigarettes per day), smoking history (years) (if applicable)	3	Fagerström Test for Nicotine Dependence (FTND) [19]	6
Alcohol consumption	Daily drinking habit (average number of units of alcohol consumed per day) and binge drinking (consuming more than 5 units of alcohol on one day)	2	Five-shot [20]	5
Nutrition intake	NutriMáx short form (a)	7	NutriMáx long form (a)	18
Perceived stress	Perceived Stress Scale (PSS-4) [21]	4	Perceived Stress Scale (PSS-10) [21]	6
*Total*		*19*		*68*

(a) NutriMáx is a customised healthy diet assessment questionnaire based on the Dutch Dietary Guidelines [22], which is used to assess diet quality and nutrition behaviour.

### Outcome measures

The primary outcome was usability of the LifeStyleScore application. Secondary outcomes were patient self-management and activation, changes in lifestyle behaviours and acceptance of the application by the HCPs.

### Usability

Usability of the LifeStyleScore application was assessed by the 10-item SUS questionnaire (Dutch version) [13]. The SUS is known as a highly robust and versatile tool for usability professionals to quickly and easily collect the user’s subjective rating of a product’s usability. The SUS scores are presented on a scale ranging from 0 to 100. A study on the SUS scores of 500 studies [23] determined that the average SUS score is 68 points. A score above 68 indicates that the design needs minor improvements or adjustments, while a score of below 68 indicates that the design might need to be re-evaluated and improved to be considered user friendly. The mean SUS score is also suitable for evaluating the usability of digital health apps [24].

### Patient activation and self-management

Patient activation was measured using the PAM®-13 questionnaire (Dutch version) [4]. The PAM-13 is a validated questionnaire that measures self-reported knowledge, skills and confidence for self-management of one’s health or chronic condition. The PAM’s algorithm produces a score along an empirical, interval level scale from 0–100 that correlates to one of four progressively higher levels of patient activation: 1) believing the patient role is important, 2) having the confidence and knowledge necessary to take action, 3) actually taking action to maintain and improve one’s health and 4) staying the course even under stress.

### Lifestyle behaviour

Changes in lifestyle behaviour were measured with the newly developed mobile and desktop LifeStyleScore application. The raw scores of the validated questionnaires used in the LifeStyleScore application were translated to a score from 1 (high risk) to 3 (low risk) per category based on the cut-off scores of the original questionnaires [17–21], Dutch Physical Guidelines [16] and Dutch Dietary Guideline [22] (see Supplement 1 of the Electronic Supplementary Material for the cut-off scores used).

### Acceptance of the application by healthcare professionals

Experiences with the application as part of the CR programme were collected via a brief focus group interview with the HCPs who were involved in patient care. The participants of the focus group session used the LifeStyleScore application and were asked to share their experience with patients using the application, whether the application was beneficial to their work, and to elaborate on how the application could be integrated further in their work and the care of patients with cardiovascular disease. Thematic analysis was used to analyse the data collected in the focus group interview.

### Sample size and statistical analysis

The sample size was selected based on expected compliance and on the ‘rule of 12’ [25] for continuous variables, which recommends to include at least 12 participants in pilot studies to provide valuable preliminary information for planning larger subsequent studies. Statistical analyses were performed using IBM SPSS for Mac (version 28.0; IBM Corporation). Descriptive statistics were used to describe the study population. The within-group changes in lifestyle behaviour were analysed using paired samples T‑tests. A McNemar test was used to compare the binary variables of patient activation levels.

## Results

### Sample characteristics and questionnaire completion

We included 43 of the 137 patients who were asked to participate. Frequently reported reasons for not participating were having already participated in studies involving questionnaires, not being proficient with mobile devices or due to comorbidities. Due to unexpected technical difficulties, 23 patients did not complete the baseline assessment or were lost to follow-up, leaving 20 patients for final analysis. Patients were mostly male (85%) and had a mean age of 61.9 ± 6.7 years, most patients were diagnosed with myocardial infarction (*n* = 16, 80%) and underwent coronary revascularisation using a percutaneous coronary intervention. Other demographic and disease characteristics are described in Tab. 3.

**Table 3:** Demographic and disease characteristics

Demographic characteristics	Total *(n)* = 20
Male (%)	17 (85%)
Age, years	61.9 ± 6.7
Height, cm	178.4 ± 10.5
Weight, kg	78.2 ± 10.9
BMI, kg/m^2^	27.4 ± 4.8
*Primary cardiac diagnosis*	
Myocardial infarction	16 (80.0%)
Unstable angina pectoris	1 (5.0%)
Stable angina pectoris	1 (5.0%)
Atrial fibrillation	2 (10.0%)
*Primary cardiac intervention*	
PCI	16 (80.0%)
CABG	2 (10.0%)
ECV	2 (10.0%)

Values are reported as *n* (%) or mean ± standard deviation.

*BMI* body mass index, *PCI* percutaneous coronary intervention, *CABG* coronary artery bypass graft surgery, *ECV* electrical cardioversion.

Of the 20 analysed patients, all participants completed the SUS and PAM-13 questionnaire at baseline and follow-up. For the baseline LifeStyleScore questionnaires, one Stage 1 alcohol questionnaire was missing, and for the follow-up questionnaires one Stage 2 physical activity questionnaire was missing. When a ‘Stage 1’ questionnaire was missing, the patient was excluded from data analysis for that specific lifestyle behaviour. When a ‘Stage 2’ questionnaire was missing, the outcomes of ‘Stage 1’ were used for data analysis.

### Usability

The mean SUS score was 69.6 ± 13.4 at baseline and 68.6 ± 15.1 at follow-up (potential score ranges from 0 to 100). This change was not statistically significant (*p* = 0.76).

### Patient activation status

**Fig. 3:**
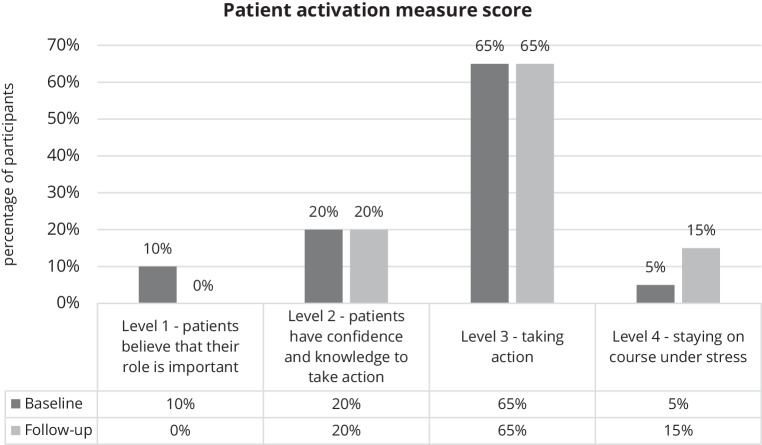
Distribution of the PAM levels at baseline and 3‑month follow-up

The mean PAM-13 score was 59.2 ± 9.5 at baseline and slightly increased to 62.0 ± 8.6 at follow-up. The change in PAM-13 scores over time was not statistically significant (*p* = 0.28). The distribution of the PAM levels is displayed in Fig. 3. The pattern of results indicates that patients moved toward taking slightly more action regarding improving their lifestyle behaviours (i.e., decreases in levels 1 and 2, and increases in levels 3 and 4), although this was not statistically significant (*p* = 0.63).

### Changes in lifestyle behaviours

**Fig. 4:**
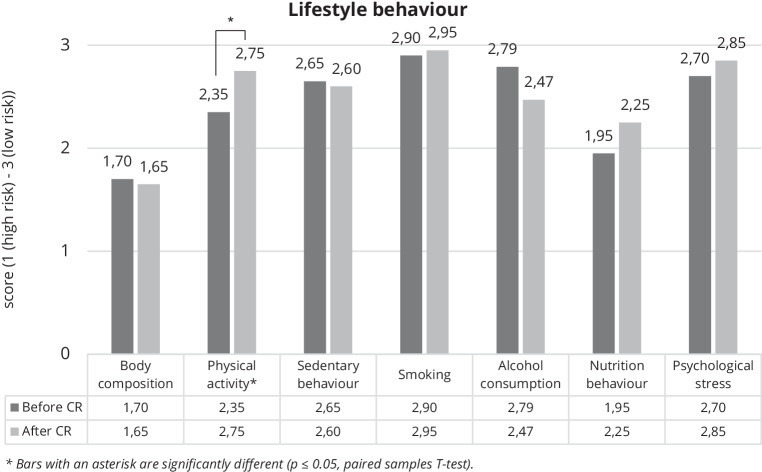
Lifestyle behaviour scores at baseline and 3‑month follow-up

The changes in cardiovascular risk behaviours are displayed in Fig. 4. Only physical activity behaviour showed a significant increase (2.4 ± 0.7 at baseline vs. 2.8 ± 0.4 after CR, *p* = 0.04). Results for the other lifestyle behaviours are described in Supplement 2 of the Electronic Supplementary Material.

### Acceptance of the application by healthcare professionals

All HCPs (*n* = 8, all case managers of the CR programme) considered the application to be a helpful tool to increase lifestyle behaviour awareness among patients, and a valuable assessment tool that could be integrated well into the CR care pathway. The HCPs indicated that their patients seemed to take action based on the use of the application and that the application is a helpful tool for patients in preparation for their consultation with their CR case manager.

## Discussion

This study shows that the usability of a novel LifeStyleScore application for comprehensive assessment of lifestyle behaviours in cardiovascular patients entering CR was satisfactory and all HCPs considered the application a useful tool for increasing lifestyle behaviour awareness. However, patient activation and most lifestyle behaviours were not significantly improved in patients who used the application for setting lifestyle goals in CR.

The LifeStyleScore application is one of the first uniform self-assessment tools based on validated instruments available for the comprehensive assessment of lifestyle behaviours in cardiovascular patients. The design and development process of the application was focused on usability, one of the key elements for successful use of applications. To our knowledge, no other studies have previously investigated the system usability of comprehensive lifestyle assessment applications used in CR, which makes it difficult to compare our findings with other studies. However, it has previously been demonstrated that the average system usability score was 68 in a total of 500 usability evaluations [23], and that this method of benchmarking is suitable for the evaluation of usability in digital health applications [24]. We can therefore conclude that the usability of the LifeStyleScore application (the mean SUS score was 69.6 ± 13.4 at baseline and 68.6 ± 15.1 at follow-up) in general is satisfactory (or ‘OK’ or ‘high marginal’ based on the adjective rating of the SUS [26]). Based on this score, we can conclude that the application needs minor improvements to move from ‘marginally acceptable’ (SUS between 50–70) to ‘acceptable’ (SUS > 71).

The use of the LifeStyleScore application as an integral part of CR was associated with a significant increase in physical activity levels, but was not associated with a significant increase in patient activation and the other lifestyle behaviours. This result may be due to the low number of assessment moments (i.e. only at baseline and at the end of the CR programme), given that frequent self-monitoring of lifestyle behaviour has been shown to be correlated with an improvement of lifestyle behaviour in other studies. For example, repeated recording (daily) of nutrition intake was shown to be associated with successful weight loss [27], and daily tracking of physical activity can increase the amount of exercise completed [27, 28]. Secondly, the application could be improved and further personalised by providing the users with more specific advice for lifestyle behaviour change, tailored to the user’s motivational level, stage of change and confidence to change, which can, for example, be profiled via validated questionnaires or by the HCPs. Finally, the implementation of e‑health applications, such as the LifeStyleScore application, in clinical settings requires careful consideration of the patient population’s demographics and technological proficiency. Some patients may require additional support to effectively use e‑health applications, while others may be resistant to the adoption of e‑health technologies [29]. As such, the development and implementation of e‑health applications should be guided by patient-centred approaches that prioritise user experience (UX) and preferences. Several UX solutions exist to make applications more interactive and engaging, which may result in more activated users. An example of an UX improvement could be to incorporate gamification techniques, such as rewards and badges, to enhance user engagement.

Further research is needed to explore the potential of the LifeStyleScore application to promote sustained behaviour change in CR patients. In particular, studies are needed to evaluate the effectiveness of the application when combined with other interventions, such as in-person counselling or group therapy, to determine the optimal approach for promoting health behaviour change in this population. Long-term follow-up studies are also necessary to assess the sustained impact of the application on patient outcomes, including changes in health behaviour and cardiovascular clinical outcomes.

### Limitations

One of the limitations of this pilot study concerns the absence of a control group. It is therefore uncertain whether changes in patient activation and lifestyle behaviours reflect the use of the LifeStyleScore application or other factors involved in CR. A second limitation of the study was the small sample size. However, the aim of the pilot study was to retrieve qualitative and quantitative feedback on the LifeStyleScore application to improve its design and test it further in larger studies. No statements can therefore be made about subpopulations (e.g., women, the elderly and patients with atrial fibrillation) which were insufficiently represented in our cohort. However, a majority of studies investigating the impact of gender (as summarised by Lewis (2018) [30]) and age on SUS ratings found no significant effect on SUS scores. And lastly, the perceived system usability of the patients was only tested quantitatively with the SUS, not with a qualitative user research method. The plan for further developing the LifeStyleScore application will include methods for collecting patient input to improve, collaboratively develop and test its next version.

## Conclusion

The LifeStyleScore application, an application for the self-assessment of cardiovascular risk behaviour, showed satisfactory usability among CR patients and its potential value was recognised by HCPs involved in CR. Yet, its use was not associated with a significant increase in patient activation and most lifestyle behaviours did not improve by using the application. These results highlight that further research is needed to find the optimal strategy to integrate lifestyle behaviour assessment tools in CR care pathways.

### Supplementary Information

Supplement 1 Questionnaires and cut-offs used in the LifeStyleScore application

Supplement 2 Paired samples T‑test of lifestyle behaviour at baseline and follow-up

## References

[1] White ND, Lenz TL, Smith K (2013). Tool guide for lifestyle behavior change in a cardiovascular risk reduction program. Psychol Res Behav Manag.

[2] De Bacquer D, Astin F, Kotseva K (2022). Poor adherence to lifestyle recommendations in patients with coronary heart disease: results from the EUROASPIRE surveys. Eur J Prev Cardiol.

[3] Brouwers RWM, Brini A, Kuijpers RWFH, Kraal JJ, Kemps HMC. Predictors of non-participation in a cardiac telerehabilitation programme: a prospective analysis. Eur Heart J Digit Health. 2022;3:81–9. 10.1093/ehjdh/ztab105 PMC9707959 36713984

[4] Hibbard JH, Mahoney ER, Stockard J, Tusler M (2005). Development and testing of a short form of the patient activation measure. Health Serv Res.

[5] Dohnke B, Nowossadeck E, Müller-Fahrnow W (2010). Motivation and Participation in a Phase III Cardiac Rehabilitation Programme: An Application of the Health Action Process Approach. Res Sports Med.

[6] Perkins S, Jenkins LS. Self-efficacy expectation, behavior performance, and mood status in early recovery from percutaneous transluminal coronary angioplasty. Heart Lung J Cardiopulm Acute Care. 1998;27:37–46. 10.1016/s0147-9563(98)90067-x 9493881

[7] Daly J, Sindone AP, Thompson DR, Hancock K, Chang E, Davidson P (2002). Barriers to Participation in and Adherence to Cardiac Rehabilitation Programs: A Critical Literature Review. Prog Cardiovasc Nurs.

[8] Balakatounis K, Angoules A, Panagiotopoulou K. Motivation for Cardiac Rehabilitation Attendance: Creating an Evidence-based. Strateg Nov Physiother. 2016;6.

[9] Russell KL, Bray SR (2010). Promoting self-determined motivation for exercise in cardiac rehabilitation: The role of autonomy support. Rehabil Psychol.

[10] Suls J, Mogavero JN, Falzon L, Pescatello LS, Hennessy EA, Davidson KW (2020). Health Behaviour Change in Cardiovascular Disease Prevention and Management: Meta-Review of Behaviour Change Techniques to Affect Self-Regulation. Health Psychol Rev.

[11] Atri SB, Sahebihagh MH, Jafarabadi MA, Behshid M, Ghasempour M, Abri F (2016). The Relationship between Health Literacy and Stages of Change in Smoking Behavior among Employees of Educational Health Centers of Tabriz University of Medical Sciences. Int J Prev Med.

[12] Walters R, Leslie SJ, Sixsmith J, Gorely T (2020). Health Literacy for Cardiac Rehabilitation: An Examination of Associated Illness Perceptions, Self-Efficacy, Motivation and Physical Activity. Int J Environ Res Public Health.

[13] Peres S, Pham T, Phillips R (2013). Validation of the System Usability Scale (SUS). Proc Hum Factors Ergon Soc Annu Meet.

[14] BRAVO-kompas [Internet]. BRAVO-Kompas. [cited 2023 Apr 18]. Available from: https://tools.kenniscentrumsportenbewegen.nl/bravo-kompas/tool/bravokompas/

[15] Brouwers R, Rongen I, Kraal J, Kemps H, van de Sande D, Vromen T. Validity and acceptance of self-assessment tools for cardiovascular risk behaviour: a systematic review [Internet]. [cited 2022 Oct 9]. Available from: https://www.crd.york.ac.uk/prospero/display_record.php?RecordID=70945

[16] Weggemans RM, Backx FJG, Borghouts L (2018). The 2017 Dutch Physical Activity Guidelines. Int J Behav Nutr Phys Act.

[17] Vandelanotte C, Bourdeaudhuij I, Philippaerts R, Sjostrom M, Sallis J (2005). Reliability and Validity of a Computerized and Dutch Version of the International Physical Activity Questionnaire (IPAQ). J Phys Act Health.

[18] TNO, Nederlands Instituut voor sport en beweging (NISB). Wat beweegt jou? Vragenlijst. 2012 [Internet]. [cited 2023 May 19]; Available from: https://publications.tno.nl/publication/100331/boxyay/tno-2012-wat.pdf

[19] Vink JM, Willemsen G, Beem AL, Boomsma DI (2005). The Fagerström Test for Nicotine Dependence in a Dutch sample of daily smokers and ex-smokers. Addict Behav.

[20] Seppä K, Lepistö J, Sillanaukee P (1998). Five-Shot Questionnaire on Heavy Drinking. Alcohol Clin Exp Res.

[21] Cohen S, Kamarck T, Mermelstein R (1983). A global measure of perceived stress. J Health Soc Behav.

[22] Looman M, Feskens EJ, de Rijk M (2015). Development and evaluation of the Dutch Healthy Diet index. Public Health Nutr.

[23] Sauro J (2011). A Practical Guide to the System Usability Scale: Background, Benchmarks & Best Practices. Measuring Usability LLC.

[24] Hyzy M, Bond R, Mulvenna M (2022). System Usability Scale Benchmarking for Digital Health Apps: Meta-analysis. JMIR Mhealth Uhealth.

[25] Moore CG, Carter RE, Nietert PJ, Stewart PW. Recommendations for Planning Pilot Studies in Clinical and Translational Research. Clin Transl Sci. 2011; 332–7. 10.1111/j.1752-8062.2011.00347.x PMC3203750 22029804

[26] Bangor A, Kortum PT, Miller JT (2008). An Empirical Evaluation of the System Usability Scale. Int J Human—computer Interact.

[27] Carels RA, Darby LA, Rydin S, Douglass OM, Cacciapaglia HM, O’Brien WH. The relationship between self-monitoring, outcome expectancies, difficulties with eating and exercise, and physical activity and weight loss treatment outcomes. Ann Behav Med Publ Soc Behav Med. 2005;30:182–90. 10.1207/s15324796abm3003_2 16336069

[28] Bravata DM, Smith-Spangler C, Sundaram V (2007). Using pedometers to increase physical activity and improve health: a systematic review. JAMA.

[29] Frishammar J, Essén A, Bergström F, Ekman T (2023). Digital health platforms for the elderly? Key adoption and usage barriers and ways to address them. Technol Forecast Soc Change.

[30] Lewis JR (2018). The System Usability Scale: Past, Present, and Future. Int J Hum-comput Int.

