# Supplementary material for: Evaluation of an application for the self-assessment of lifestyle behaviour in cardiac patients
Source: Neth Heart J. 2023 Dec 7;32(1):55–62. doi: 10.1007/s12471-023-01835-7 (PMC10781924; doi:10.1007/s12471-023-01835-7)
Supplement: Supplementary file 1 — Supplement 1 Questionnaires and cut-offs used in the LifeStyleScore application [file 12471_2023_1835_MOESM1_ESM.docx]

**Supplement 1** Questionnaires and cut-offs used in the LifeStyleScore application

| **Body composition** | |
| --- | --- |
| **Stage 1 (only 1 stage)** | |
| **Questions asked:**   1. Gender (m/f) 2. Age (years) 3. Length (cm) 4. Weight (kg) 5. Waist circumference (cm) | |
| **Cut-offs** | |
| **Low risk  (3 – Green)** | BMI < 25 kg/m^2^ AND waist circumference of < 94 cm (male) or 80 cm (female) |
| **Medium risk  (2 – Yellow)** | BMI 25 - 29,9 kg/m^2^ AND/OR waist circumference of < 94 – 102 cm (male) or 80 – 88 (female) |
| **High risk  (1 – Red)** | BMI ≥ 30 kg/m^2^ AND/OR waist circumference of > 102 cm (male) or > 88 (female) |

| **Physical activity and sedentary behaviour** | | |
| --- | --- | --- |
| **Stage 1 (physical activity and sedentary behaviour are combined in Stage 1)** | | |
| **Questions asked:**   1. Number of days per week performing 30 min of moderate intensity physical activities 2. Number of days per week performing vigorous intensity physical activities 3. How many hours per day spent sitting (on average) a. 0 – 4 hours b. 4 – 8 hours c. > 8 hours 4. How many breaks taken while sitting  a. Once per 30 minutes b. Once per hour c. Once per two hours or less | | |
| **Cut-offs – Physical activity (only question 1 and 2)** | | **Action** |
| **Low risk  (3 – Green)** | If Q1 is answered with > 4 days AND Q2 is answered with > 1 day | End questionnaire subset |
| **Medium risk  (2 – Yellow)** | If Q1 is answered with > 4 days OR Q2 is answered with > 2 days | Continue to Stage 2 (Physical activity) |
| **High risk  (1 – Red)** | All else lower than the cut-offs described in Medium and Low risk. | Continue to Stage 2 (Physical activity) |
| **Cut-offs – Sedentary behaviour (only question 3 and 4)** | | **Action** |
| **Low risk  (3 – Green)** | 0 – 4 hours per day spent sitting (a) AND one break per 30 minutes of sitting (a) | End questionnaire subset |
| **Medium risk  (2 – Yellow)** | > 4 hours of sitting (b-c) AND/OR one break per hour of sitting or less | Continue to Stage 2 (Sedentary behaviour) |
|  | | |
| **Stage 2 (Physical activity)** | | |
| Validated questionnaire: International Physical Activity Questionnaires (IPAQ)[1]. Calculation of score and categories of the original questionnaire are used [2]. | | |
| **Cut-offs** | | |
| **Low risk  (3 – Green)** | IPAQ category 3 – High level of physical activity | |
| **Medium risk (2 – Yellow)** | IPAQ category 2 – Moderate level of physical activity | |
| **High risk  (1 – Red)** | IPAQ category 1 – Low level of physical activity | |
|  | | |
| **Stage 2 (Sedentary behaviour)** | | |
| Validated questionnaire: ‘Wat beweegt jou’ (only the ‘Hoeveel zit jij’ - section)[3].  Calculation of score and categories of the original questionnaire are used. | | |
| **Cut-offs – ‘Wat beweegt jou’** | | |
| **Low risk  (3 – Green)** | Category 3 – ‘Je zit niet veel!’ (English translation: ‘You do not sit too much’) | |
| **Medium risk  (2 – Yellow)** | Category 2 – ‘Je zit niet te veel, maar kunt nog minderen!’ (English translation: ‘You do not sit too much, but you can sit less’) | |
| **High risk  (1 – Red)** | Category 1 – ‘Je zit te veel!’ (English translation: ‘You sit too much’) | |

| **Smoking cessation** | | |
| --- | --- | --- |
| **Stage 1** | | |
| **Questions asked:**   1. Do you smoke? (yes/no) 2. Have you smoked in the past? (yes/no) 3. (if question 1 is equal to ‘Yes’) Are you planning to stop smoking soon? (yes/no) | | |
| **Cut-offs** | | **Action** |
| **Low risk  (3 – Green)** | If Q1 is answered with ‘No’. OR If Q1 is answered with ‘Yes’ AND Q3 is answered with ‘Yes’ | End questionnaire subset |
| **High risk  (1 – Red)** | If Q1 is answered with ‘Yes’ and Q3 is answered with ‘No’ | Continue to Stage 2 |
|  | | |
| **Stage 2** | | |
| Validated questionnaire: Fagerstrom Test for Nicotine Dependence (FTND)[4].  Cut-off scores are based on the original questionnaire. | | |
| **Cut-offs** | | |
| **Low risk  (3 – Green)** | Low level of nicotine dependence (score 1-2) | |
| **Medium risk  (2 – Yellow)** | Moderate level of nicotine dependence (score 3-6) | |
| **High risk  (1 – Red)** | High level of nicotine dependence (score > 7) | |

| **Alcohol consumption** | | |
| --- | --- | --- |
| **Stage 1** | | |
| **Questions asked:**   1. In the past 30 days, have you consumed more than 5 units of alcohol on one night? (yes/no) 2. How many units of alcohol do you consume on average per day? | | |
| **Cut-offs** | | **Action** |
| **Low risk  (3 – Green)** | If Q1 is answered with ‘No’. AND  If Q2 is answered with 1 or less units of alcohol on average per day | End questionnaire subset |
| **High risk  (1 – Red)** | If Q1 is answered with ‘Yes’. OR If Q2 is answered with > 1 units of alcohol on average per day | Continue to Stage 2 |
|  | | |
| **Stage 2** | | |
| Validated questionnaire: Five-shot questionnaire [5]. Calculation of score and interpretation of the original questionnaire are used. | | |
| **Cut-offs** | | |
| **Low risk  (3 – Green)** | Five-shot score less than 2.5. | |
| **Medium risk  (2 – Yellow)** | Five-shot score of 2.5 or greater, as this indicates possible alcohol misuse. | |

| **Nutrition intake** | | | |
| --- | --- | --- | --- |
| **Stage 1** | | | |
| Custom questionnaire: NutriMáx Short-Form **Questions asked in the following food categories:**   1. Vegetables 2. Fruit 3. Nuts 4. Whole-wheat products 5. Fats 6. Fish 7. Legumes   0 or 1 point can be scored per question. | | | |
| **Cut-offs** | | | **Action** |
| **Low risk  (3 – Green)** | ≥ 6 points | | End questionnaire subset |
| **Medium risk  (2 – Yellow)** | 4 – 5 points | | Continue to Stage 2 |
| **High risk  (1 – Red)** | ≤ 3 points | | Continue to Stage 2 |
|  | | | |
| **Stage 2** | | | |
| Custom questionnaire: NutriMáx Long-Form  **Questions asked in the following food categories:**   1. Vegetables 2. Fruit 3. Whole-wheat products 4. Nuts 5. Dairy 6. Tea 7. Legumes 8. Fish 9. Fats 10. Coffee 11. Meat 12. Sugary drinks 13. Alcohol 14. Salt 15. Snacks 16. Ready-made meals   0 to 2 points can be scored per question. Max scores can be 28, 30 or 32 based on given answers. The max scores depend on whether the questions related ‘Coffee’ and ‘Meat’ are answered with ‘Yes’ or ‘No’ (If ‘Coffee’ and ‘Meat’ are both answered with ‘Yes’, the max score is 32. If ‘Coffee’ or ‘Meat’ is answered with ‘Yes’ and the other one with ‘No’, the max score is 30. If ‘Coffee’ and ‘Meat’ are both answered with ‘No’, the max score is 28.) | | | |
| **Cut-offs** | | | |
|  | **Max 28** | **Max 30** | **Max 32** |
| **Low risk  (3 – Green)** | ≥ 24 | ≥ 26 | ≥ 28 |
| **Medium risk  (2 – Yellow)** | 16 - 23 | 18 - 25 | 20 - 27 |
| **High risk  (1 – Red)** | < 16 | < 18 | < 20 |

| **Perceived stress** | | |
| --- | --- | --- |
| **Stage 1** | | |
| Validated questionnaire: 4-item Perceived Stress Scale (PSS-4)[6]. Cut-off scores are based on the original questionnaire. | |  |
| **Cut-offs** | | **Action** |
| **Low risk  (3 – Green)** | Score ≤ 4 | End questionnaire subset |
| **Medium risk  (2 – Yellow)** | Score 5 – 8 | Continue to Stage 2 |
| **High risk  (1 – Red)** | Score > 8 | Continue to Stage 2 |
|  | | |
| **Stage 2** | | |
| Validated questionnaire: 10-item Perceived Stress Scale (PSS-10)[6]. Calculation of score and categories of the original questionnaire are used. | | |
| **Cut-offs** | | |
| **Low risk  (3 – Green)** | PSS-10 category 3 – Low perceived stress (score 0 – 13) | |
| **Medium risk  (2 – Yellow)** | PSS-10 category 2 – Moderate perceived stress (score 14 – 16) | |
| **High risk  (1 – Red)** | PSS-10 category 1 – High perceived stress (score 27 -40) | |

**References**

1. Vandelanotte C, Bourdeaudhuij I, Philippaerts R, Sjostrom M, Sallis J. Reliability and Validity of a Computerized and Dutch Version of the International Physical Activity Questionnaire (IPAQ). J Phys Act Health. 2005;2:63–75.

2. Sjostrom, M., Ainsworth, B.E., Bauman, A., Bull, F.C., Hamilton-Craig, C.R., & Sallis, J.F. (2005). Guidelines for data processing analysis of the International Physical Activity Questionnaire (IPAQ) - Short and long forms.

3. TNO, Nederlands Instituut voor sport en beweging (NISB). Wat beweegt jou? Vragenlijst. 2012 [Internet]. [cited 2023 May 19]; Available from: https://publications.tno.nl/publication/100331/boxyay/tno-2012-wat.pdf

4. Vink JM, Willemsen G, Beem AL, Boomsma DI. The Fagerström Test for Nicotine Dependence in a Dutch sample of daily smokers and ex-smokers. Addict Behav. 2005;30:575–9.

5. Seppä K, Lepistö J, Sillanaukee P. Five-Shot Questionnaire on Heavy Drinking. Alcohol Clin Exp Res. 1998;22:1788–91.

6. Cohen, S., Kamarck, T., & Mermelstein, R. (1983). A global measure of perceived stress. Journal of Health and Social Behavior, 24, 385-396.
