# Supplementary material for: Evaluation of an application for the self-assessment of lifestyle behaviour in cardiac patients
Source: Neth Heart J. 2023 Dec 7;32(1):55–62. doi: 10.1007/s12471-023-01835-7 (PMC10781924; doi:10.1007/s12471-023-01835-7)
Supplement: Supplementary file 2 — Supplement 2 Paired samples T‑test of lifestyle behaviour at baseline and follow-up [file 12471_2023_1835_MOESM2_ESM.docx]

**Supplement 2** Paired samples T-test of lifestyle behaviour at baseline and follow-up

| ***Lifestyle domain*** | ***Baseline score***  ***(****mean ± standard deviation)* | ***Follow-up score***  ***(****mean ± standard deviation)* | ***df*** | ***Two-Sided p*** |
| --- | --- | --- | --- | --- |
| Body composition | 1.70 ± 0.66 | 1.65 ± 0.75 | 19 | 0.716 |
| Physical activity | 2.35 ± 0.75 | 2.75 ± 0.44 | 19 | 0.042* |
| Sedentary behaviour | 2.65 ± 0.49 | 2.60 ± 0.50 | 19 | 0.666 |
| Smoking cessation | 2.90 ± 0.31 | 2.95 ± 0.22 | 19 | 0.330 |
| Alcohol consumption | 2.79 ± 0.63 | 2.47 ± 0.90 | 18(a) | 0.083 |
| Nutrition intake | 1.95 ± 0.89 | 2.25 ± 0.79 | 19 | 0.055 |
| Perceived stress | 2.70 ± 0.47 | 2.85 ± 0.37 | 19 | 0.186 |
| (a) One participant did not complete the alcohol consumption questionnaire, as a result the data analysis could only be executed for 19 participants in this lifestyle domain.  * Numbers with an asterisk are significantly different (p ≤ 0.05, paired samples T-test). | | | | |
